# Bioactivities and Extract Dereplication of *Actinomycetales* Isolated From Marine Sponges

**DOI:** 10.3389/fmicb.2019.00727

**Published:** 2019-04-09

**Authors:** José D. Santos, Inês Vitorino, Mercedes De la Cruz, Caridad Díaz, Bastien Cautain, Frederick Annang, Guiomar Pérez-Moreno, Ignacio Gonzalez Martinez, Jose R. Tormo, Jesús M. Martín, Ralph Urbatzka, Francisca M. Vicente, Olga M. Lage

**Affiliations:** ^1^Department of Biology, Faculty of Sciences, University of Porto, Porto, Portugal; ^2^Interdisciplinary Centre of Marine and Environmental Research, University of Porto, Matosinhos, Portugal; ^3^Fundación MEDINA, Centro de Excelencia en Investigación de Medicamentos Innovadores en Andalucía, Parque Tecnológico de Ciencias de la Salud, Granada, Spain; ^4^Instituto de Parasitología y Biomedicina “López-Neyra”, Consejo Superior de Investigaciones Científicas (CSIC), Granada, Spain

**Keywords:** *Actinomycetales*, antimicrobials, anti-cancer, anti-parasitic, anti-obesogenic, marine sponges

## Abstract

In the beginning of the twenty-first century, humanity faces great challenges regarding diseases and health-related quality of life. A drastic rise in bacterial antibiotic resistance, in the number of cancer patients, in the obesity epidemics and in chronic diseases due to life expectation extension are some of these challenges. The discovery of novel therapeutics is fundamental and it may come from underexplored environments, like marine habitats, and microbial origin. *Actinobacteria* are well-known as treasure chests for the discovery of novel natural compounds. In this study, eighteen *Actinomycetales* isolated from marine sponges of three *Erylus* genera collected in Portuguese waters were tested for bioactivities with the main goal of isolating and characterizing the responsible bioactive metabolites. The screening comprehended antimicrobial, anti-fungal, anti-parasitic, anti-cancer and anti-obesity properties. Fermentations of the selected strains were prepared using ten different culturing media. Several bioactivities against the fungus *Aspergillus fumigatus*, the bacteria *Staphylococcus aureus* methicillin-resistant (MRSA) and the human liver cancer cell line HepG2 were obtained in small volume cultures. Screening in higher volumes showed consistent anti-fungal activity by strain *Dermacoccus* sp. #91-17 and *Micrococcus luteus* Berg02-26. *Gordonia* sp. Berg02-22.2 showed anti-parasitic (*Trypanosoma cruzi*) and anti-cancer activity against several cell lines (melanoma A2058, liver HepG2, colon HT29, breast MCF7 and pancreatic MiaPaca). For the anti-obesity assay, *Microbacterium foliorum* #91-29 and #91-40 induced lipid reduction on the larvae of zebrafish (*Danio rerio*). Dereplication of the extracts from several bacteria showed the existence of a variety of secondary metabolites, with some undiscovered molecules. This work showed that *Actinomycetales* are indeed good candidates for drug discovery.

## Introduction

The technological advances made in the twenty and twenty-first centuries gave rise to the most prosperous society which has ever existed (International Monetary Fund Research Department [IMF], 2000). This well-being is associated with an overall drastic increase in the average life expectancy (Vaupel, 2010) and with several problems faced by the today’s society. One of these problems is a result of the increase in caloric uptake and lack of exercise which results in an epidemic of overweight. Obese people present major challenges, as obesity entails a myriad of risk factors for chronic diseases like diabetes, heart diseases and even some cancers (Hruby and Hu, 2015). Furthermore, the extended life span associated with environmental factors such as tobacco smoking, industrialization and urbanization are causes behind the increase in cancer incidence rates (You and Henneberg, 2018). Antibiotic resistance is another emerging problem. Overabundance in the use of antibiotics as well as their intense misuse resulted in the phenomenon of antibiotic resistance, which is rapidly occurring worldwide, with many commonly used antibiotics having already been rendered useless (Ventola, 2015). Several are thus the challenges at the beginning of the twenty-first century.

In recent years, research into new compounds has been focused in the ocean and many marine organisms are proving to be good sources of interesting new leads (Imhoff et al., 2011) Drugs like xestospongin C and several manoalides were discovered in marine animals like sponges (Miyamoto et al., 2000; Stowe et al., 2011). Sponges have a diverse microbiological community, often showing the presence of archaea, fungi, microalgae and a great diversity of bacterial phyla (Taylor et al., 2007). While sponges are good candidates for drug discovery, a major setback is that sponges may only contain minute quantities of these compounds in their body, which may invalidate clinical trials (Mehbub et al., 2014). However, it seems that the symbiotic microbiological community is the true origin of several relevant compounds (Yoo Kyung et al., 2001). It is also known that *Actinobacteria* are copious producers of bioactive metabolites. Particularly prolific are species isolated from soil and affiliated to the *Actinomycetales*, most notably the genus *Streptomyces* (Miao and Davies, 2010). Less studied are marine *Actinobacteria* which have nonetheless already shown potential as sources for novel leads. Several bioactive molecules, ranging from antimalarians like salinipostins, cytotoxics such as marinomycins to antibacterials as abyssomicins, have all been isolated from *Actinobacteria* found in marine environments (Bister et al., 2004; Kwon et al., 2006; Schulze et al., 2015; Dhakal et al., 2017).

This study aimed to contribute to the need of finding new and more effective bioactive molecules against several of the earlier mentioned threats faced by human kind nowadays. For this, eighteen species from the *Actinomycetales* previously isolated from *Erylus* spp. sponges collected in Portuguese marine waters (Açores, Madeira, and continental shelf) were screened for antimicrobial, anti-cancer, anti-parasitic and anti-obesogenic activities.

## Materials and Methods

### Biological Material

The bacteria under study belong to the order *Actinomycetales* within the phylum *Actinobacteria* and were isolated from marine sponges of the genus *Erylus, E. discophorus* (Berg01 and Berg02, from the continental shelf at Berlengas, Portugal) and *E. deficiens* (#91, from the continental shelf at Gorringe, Portugal) and *E. mamillaris* (SM, Açores, Portugal) (Table 1). Most of these strains showed the presence of interesting secondary metabolism genes and/or bioactivities in previous antimicrobial screenings (Table 1; Graca et al., 2013, 2015).

**Table 1:** List of *Actinomycetales* used in this study evidencing their physiological affiliation and bioactive potential.

Strain ID	Affiliation	Secondary metabolism gene	Previous bioactivity
#91_17^∗^	*Dermacoccus* sp. Ellin185; AF409027	NRPS	CA
#91_20	*Rhodococcus hoagii* CUB1156; AJ272469	N/D	NA
#91_29^∗^	*Microbacterium foliorum* BJC15-C14; JX464206	N/D	CA
#91_31^∗^	*Microbacterium hydrocarbonoxydans* 3084; EU714352	N/D	CA
#91_34^∗^	*Microbacterium esteraromaticum* 2122; EU714337	N/D	CA
#91_35^∗^	*Microbacterium phyllosphaerae (T)*; DSM 13468; P 369/06; AJ277840	PKS-I	CA; VA
#91_36.1^∗^	*Rhodococcus equi* type strain*:* DSM20307; X80614	PKS-I; NRPS	CA; VA; EC
#91_37^∗^	*Microbacterium foliorum* BJC15-C1; JX401513	N/D	CA; VA
#91_40^∗^	*Microbacterium foliorum* BJC15-C14; JX464206	PKS-I	CA; VA
#91_44	*Rhodococcus* sp.	PKS-I; NRPS	NA
#91_54	*Rhodococcus qingshengii* KUDC1814; KC355321	NRPS	CA, VA
SM 115	*Agrococcus baldri* B-G-NA10	PKS-I	NA
SM 116	*Agrococcus baldri* B-G-NA10	PKS-I	NA
Berg01-119c	*Microbacterium sp.* ZJY-409	N/D	VA; VF
Berg02-22.2^∗^	*Gordonia sp.* DEOB200; AY927227	PKS-I	BS
Berg02-26	*Micrococcus luteus;* KCL-1; DQ538135	N/D	NA
Berg02-78^∗^	*Gordonia terrae* 3269aBRRJ; FJ200386	PKS-I; NRPS	BS
Berg02-79^∗^	*Microbacterium sp.* M63-2; EF061897	N/D	BS


*^∗^Strains assayed for anti-obesity; PKS-I, Polyketide Synthase I; NRPS, Non-ribosomal Peptide Synthetases; N/D, Not determined; NA, No Activity; CA, Candida albicans; BS, Bacillus subtilis; EC, Escherichia coli; VA, Vibrio anguillarum; VF, Vibrio fisheri*.

For the antimicrobial assays six different pathogens were tested, *Escherichia coli* ATCC25922 (EcoWT), *Klebsiella pneumoniae* ATCC 700603, Methicillin-resistant *Staphylococcus aureus* MB5393 (MRSA), *Staphylococcus aureus* ATCC29213 (MSSA), *Acinetobacter. baumannii* MB5973 *Aspergillus fumigatus* ATCC46645, and *Candida albicans* ATCC64124. For the anti-cancer assays the following cell lines were tested: human melanoma (A2058), human lung carcinoma (A549), human hepatocellular carcinoma (HepG2), human colon cancer (HT29), human breast cancer (MCF7), and human pancreatic cancer (MIA PaCa-2). Anti-parasitic activity was assessed using the parasite *Trypanosoma cruzi* Tulahuen C4 strain. As *T. cruzi* is an obligate intracellular parasite, it was cultivated inside host L6 rat skeletal muscle cells. Anti-obesogenic activity was tested using the zebrafish (*Danio rerio*) larvae.

### Growth Media

The various *Actinomycetales* were cultivated and maintained in Marine Agar (MA) medium (Graca et al., 2013, 2015) at 25°C in darkness.

To conduct the screenings, liquid fermentations of the strains were prepared, using ten different culturing media. These different media provide a range of nutritional conditions (from oligotrophic to heterotrophic status) aiming to favor the production of bioactive metabolites. These include several ready prepared media such as Antibiotic Broth (AB) (1.0 g/L dextrose, 3.68 g/L K_2_HPO_4_, 1.5 g/L meat extract, 5.0 g/L peptone, 1.32 g/L KH_2_PO_4_, 3.5 g/L NaCl and 1.5 g/L yeast extract), Tryptic Soy Broth (TSB) (17 g/L tryptone, 3 g/L phytone, 5 g/L NaCl, 2.5 g/L K_2_HPO_4_ and 2.5 g/L glucose) and Marine Broth (MB) (5 g/L peptone, 1 g/L yeast extract, 40 g/L sea salts) CGY (Rojas et al., 2009), DEF-15 (Lam et al., 1995), IN-CRY (Obata et al., 1999), R358 (Jensen et al., 2007), and other media specified in Table 2. Moreover, as all the strains were previously isolated from a marine environment, Sea salts (Sigma-Aldrich) were added to all the media at a concentration of 30 g/L, except in the medium MB.

**Table 2:** Composition of the media used for liquid culture extractions.

Reagents (g/L)	Medium		
	
	FPY-12	M016	R2A
Yeast Extract	–	1.0	5.0^∗^ 10^-1^
Casein hydrolysate	–	–	5.0^∗^ 10^-1^
Glucose	10	10	5.0^∗^ 10^-1^
Fructose	20	–	–
Maltose	10	10	–
Peptone	5	–	5.0^∗^ 10^-1^
Amicase	5	–	–
Starch	–	10	5.0^∗^ 10^-1^
Soytone	–	5.0	–
Tryptone	–	4.0	–
K_2_HPO_4_	–	2.0^∗^ 10^-1^	3.0^∗^ 10^-1^
NaCl	–	2.0^∗^ 10^-2^	–
KCl	–	2.0^∗^ 10^-5^	–
MgSO_4_.7H_2_O	–	5.0^∗^ 10^-2^	4.0^∗^ 10^-2^
KH_2_PO_4_	–	1.0^∗^ 10^-1^	–
CaCl_2_.H_2_O	–	5.0^∗^ 10^-2^	–
C_3_H_3_NaO_3_	–	–	3.0^∗^ 10^-1^
FeSO_4_.7H_2_O	5.0^∗^ 10^-4^	–	–
ZnCl_2_	–	2.0^∗^ 10^-5^	–
ZnSO_4_.7H_2_O	5.0^∗^ 10^-4^	–	–
MnSO_4_.H_2_O	1.0^∗^ 10^-4^	1.0^∗^ 10^-4^	–
CuSO_4_.5H_2_O	5.0^∗^ 10^-5^	–	–
CoCl_2_.6H_2_O	5.0^∗^ 10^-5^	2.0^∗^ 10^-5^	–
SnCl_2_.2H_2_O	–	5.0^∗^10^-6^	–
H_3_BO_3_	–	1.0^∗^ 10^-5^	–
Na_2_MoO_4._2H_2_O	–	1.2^∗^ 10^-5^	–
CuSO_4_	–	1.5^∗^ 10^-5^	–
FeCl_3_	–	5.8^∗^ 10^-3^	–


### Extraction Protocols

#### Small Scale-Culture Extraction

A first small-scale fermentation in 0.8 mL cultures was performed. Bacterial strains were grown in the 10 media in 96-well plates (Duetz system ^1^) (Duetz et al., 2000; Minas et al., 2000; Duetz, 2007; Palomo et al., 2013; Pan et al., 2019) for 5 days at 25°C, 20% humidity and 300 revolutions per minute (rpm). After incubation, 0.8 mL acetone were added to each bacterial culture. The acetone/culture mixture was mixed for 1 h at 170 rpm and later evaporated up to a final volume of 0.6 mL in a Genevac^®^ centrifugal evaporator. Due to a large presence of salts that might influence subsequent assays, the 0.6 mL were captured in a Waters^TM^ Oasis^®^ HLB extraction plates, with the sorbent Oasis^®^ HLB. The Oasis^®^ HLB plate was equilibrated using first methanol and after HPLC grade water. The extract was run though the Oasis^®^ HLB plate in 0.2 mL steps, vacuum-eluted and the collected volume discarded. Subsequently, the secondary metabolites were captured with methanol. The methanol was, then removed in a Genevac^®^ and the recovered metabolites were dissolved in 0.4 mL 20% DMSO. This allowed a twofold concentration increase of the extracts. Additionally, as controls, culture media were also extracted with the same protocol.

#### Medium-Scale Culture Extraction

Bacteria that showed bioactive extracts were re-fermented in higher volumes (EPA vials system – 40 mL). Ten milliliters of bacterial cultures were grown for 5 days at 25°C with 20% humidity and 220 rpm. After the 5 days period, 10 mL acetone were added (1:1) to each culture and mixed for 1 h. The acetone/culture mixture was then evaporated under nitrogen to 9 milliliters to remove all traces of acetone. To remove the salt contamination, the resin SEPABEADS^®^ SP207ss was used. Two milliliters of a ready prepared suspension of SP207ss were added to the culture and mixed for 1 h. The vials were then centrifuged, and the supernatant discarded. The resin was washed with HPLC water. Ten mL of acetone were added to the resin, centrifuged and the acetone was collected, evaporated under nitrogen and dissolved in 2 mL of 20% DMSO. A fivefold increase of the extracts was achieved. As controls, media samples were also extracted with the same protocol.

#### Anti-obesogenic Assays Culture Extraction

To test possible anti-obesity compounds produced by the selected strains, each strain was grown in 100 mL Marine Broth at 25°C, 200 rpm for 5 days. For the extraction, cell pellets were lyophilized and mixed twice for 30 min with 50 mL methanol. The collected methanol was dried in a rotatory evaporator and the weight of the extract measured. The extracts were stored at -20°C and, when needed, dissolved in 100% DMSO at a concentration of 10 mg/mL.

### Bioactivity Assays

#### Antimicrobial Assays

Previously described methods using pathogenic microorganisms from Fundación MEDINA’s collection were performed to test for antibacterial and antifungal properties (Monteiro et al., 2012; Audoin et al., 2013). Briefly, single colonies of each microorganism were incubated overnight at 37°C and 220 rpm in their corresponding medium and then diluted in order to obtain assay inoculum of approximately 1.1 × 10^6^ CFU/mL for methicillin-resistant *S. aureus* MB 5393, 5.0 × 10^5^ CFU/mL for *A. baumannii* MB5973, *E. coli* ATCC 25922, *K. pneumoniae* ATCC 700603 and *S. aureus* ATCC 29213 and 2.5 × 10^4^ CFU/mL for *A. fumigatus* ATCC46645. For *C. albicans*, the OD at 660 nm of the liquid culture was adjusted to 0.25 and diluted 1:100 for assay inoculum.

For all the assays 90 μL/well of the corresponding diluted inoculum were mixed with 10 μL/well of extract. Positive and negative internal plate controls were included following the previously described methodologies. Absorbance or fluorescence were measured with an Envision plate reader. Genedata Screener software (Genedata, Inc., Basel, Switzerland) was used to analyze the data and to calculate the percentage of growth inhibition of the extracts and the RZ’ factor to estimate the robustness of the assays (Zhang et al., 1999). In all experiments performed in this work, the RZ’ factor obtained was between 0.87 and 0.92.

#### Anti-cancer Assays

The cytotoxic bioactivity of the extracts was tested by using the portfolio of HTS assays from MEDINA according to Cautain et al. (2015). Cytotoxic activity of the extracts (5 μL in 19 μL medium) was tested against human hepatocellular carcinoma (HepG2) for Duetz extracts and against human melanoma (A2058), human lung carcinoma (A549), human hepatocellular carcinoma (HepG2), human colon cancer (HT29), human breast cancer (MCF7), and human pancreatic cancer (MIA PaCa-2) for EPA vials extracts.

#### Anti-parasitic Assays

Anti-parasitic bioactivity of the extracts (5 μL of 1:1500 dilution) was tested against *T. cruzi* Tulahuen C4 strain according to Annang et al. (2015).

#### Anti-obesogenic Assays

The anti-obesity activity of the extracts taken from 11 strains arbitrarily chosen (Table 1) was tested using zebrafish larvae as described in Urbatzka et al. (2018). Briefly, hatched larvae were transferred to 48-well plates, 6–8 individual larvae per well, with 750 μL water and N-phenylthiourea. Larvae were then treated with 10 μg/mL extracts or 0.1% DMSO or 50 μM Resveratrol for 48 h. At 24 h, the solutions in the wells were renewed and 10 ng/ml Nile red added. For imaging, the larvae were anesthetized with 0.03% tricaine, fluorescence intensity acquired in a fluorescence microscope (Leica DM6000B) and the images analyzed with the software ImageJ. Statistical significance was evaluated with an ANOVA and Dunnett’s test.

#### Dereplication

Dereplication of the extracts was performed by Liquid Chromatography/High-Resolution Mass Spectroscopy (LC/HRMS) which was performed in an Agilent 1200 Rapid Resolution HPLC interfaced to a Bruker maXis mass spectrometer. The column used for separation was a Zorbax SB-C8 column (2.1 × 30 mm, 3.5 mm particle size), with two solvents used for the mobile phase. Both solvents were composed of water and acetonitrile in a 90:10 ratio for solvent A and in a 10:90 ratio for B. Both solvents contained 13 mM ammonium formiate and 0.01% trifluoracetic acid. The mass spectrometer was operated in positive ESI mode. The retention time and exact mass of the components were compared against Fundación MEDINA’s high resolution mass spectrometry database, and when a match was obtained it was reported as a named compound. For the components with no matches in the MEDINA database, the predicted molecular formula and exact mass were searched for in the Chapman and Hall Dictionary of Natural Products database. If a plausible match was found, considering the exact mass/molecular formula, the producing microorganism and the target assay, the molecule was reported as a suggested component of the fraction (Perez-Victoria et al., 2016).

## Results

Small-scale extracts (0.8 mL cultures, performed in Duets plates) are useful for a rapid evaluation of the bioactive profile of the strains. However, due to the small volume of the extracts, only a limited number of assays could be performed. For this reason, only the antimicrobial and anti-parasitic assays were run with the full number of targets, while in the anti-cancer assay, only the HepG2 cell line was assayed. This cell line is used as a model system for studies of liver toxicity (Mersch-Sundermann et al., 2004) which is an important characteristic of the drugability of any molecule.

From the 18 different strains tested in the 10 media, only 6 strains demonstrated bioactivity in one or more of the assays performed (Table 3). Antifungal activity against *A. fumigatus* ATCC46645 was obtained in several media with strains #91_17, Berg02-26, Berg02-22.2, and Berg02-78. Strain Berg02-79 showed activity against the methicillin-resistant *S. aureus* (MRSA) and strains #91_40, Berg02-22.2 Berg02-78 and Berg02-79 were effective against HepG2 cells. Berg02-78 extracts proved to be bioactive against *T. cruzi* Tulahuen C4. As extracts from strains #91_40, Berg02-22.2, Berg02-78, and Berg02-79 in medium IN-CRY proved to be hepatotoxic, no further testing was done with these extracts.

**Table 3:** Summary of the bioactivities obtained with the Duetz extracts.

Strain ID	Affiliation	Bioactivity		
		
		Target	Medium	% inhibition or death
#91_17	*Dermacoccus* sp.	AF	CGY	66
			IN-CRY	86
			M016	60
#91_40	*Microbacterium foliorum*	HepG2	IN-CRY	66
Berg02-22.2	*Gordonia* sp.	AF	AB	64
		HepG2	IN-CRY	62
Berg02-26	*Micrococcus luteus*	AF	IN-CRY	69
			R358	57
Berg02-78	*Gordonia terrae*	AF	AB	51
		TC	IN-CRY	50
Berg02-79	*Microbacterium* sp.	MRSA	R358	100
		HepG2	IN-CRY	56


*AF, A. fumigatus ATCC46645; CA, C. albicans ATCC64124; MRSA, Methicillin-resistant S. aureus MB 5393; TC, T. cruzi Tulahuen C4*.

With the medium scale extracts (10 mL culture – EPA vials) and as the volume was no longer a limitation, all microbiological, parasitic and cancer cell lines targets and assays were tested. Antifungal activity against *A. fumigatus* ATCC46645 and *C. albicans* ATCC64124 was obtained with strains #91_17 (in media CGY and M016) and Berg02-26 (in medium IN-CRY) (Table 4). Regarding the anticancer assays, the extracts from AB medium of strain Berg02-22.2 showed activity against human melanoma cell line (A2058), human hepatocellular carcinoma cell line (HepG2), human colon cancer cell line (HT29), human breast cancer cell line (MCF7), and human pancreatic cancer cell line (MIA PaCa-2) (Table 4). No activity was observed against the human lung carcinoma cell line (A549). Furthermore, strain Berg02-22.2 also showed anti-parasitic activity against *T. cruzi* Tulahuen C4 (Table 4). With the medium scale extracts, the bioactivities previously detected in the small-scale extracts of strains Berg02-78 and Berg02-79 were not confirmed (Tables 3, 4).

**Table 4:** Summary of the bioactivities obtained with the EPA extracts.

Strain ID	Affiliation	Bioactivity		
		
		Target	Medium	% inhibition or death
#91_17	*Dermacoccus* sp.	AF	CGY	67
			M016	93
		CA	CGY	82
			M016	100
Berg02-22.2	*Gordonia* sp.	TC	AB	91
		A2058	AB	71
		HepG2	AB	88
		HT29	AB	78
		MCF7	AB	82
		MIAPaCa-2	AB	72
Berg02-26	*Micrococcus luteus*	AF	IN-CRY	58
		CA	IN-CRY	75


*AF, A. fumigatus ATCC46645; CA, C. albicans ATCC64124; MRSA, Methicillin-resistant S. aureus MB 5393; TC, T. cruzi Tulahuen C4.*

**FIGURE 1:**
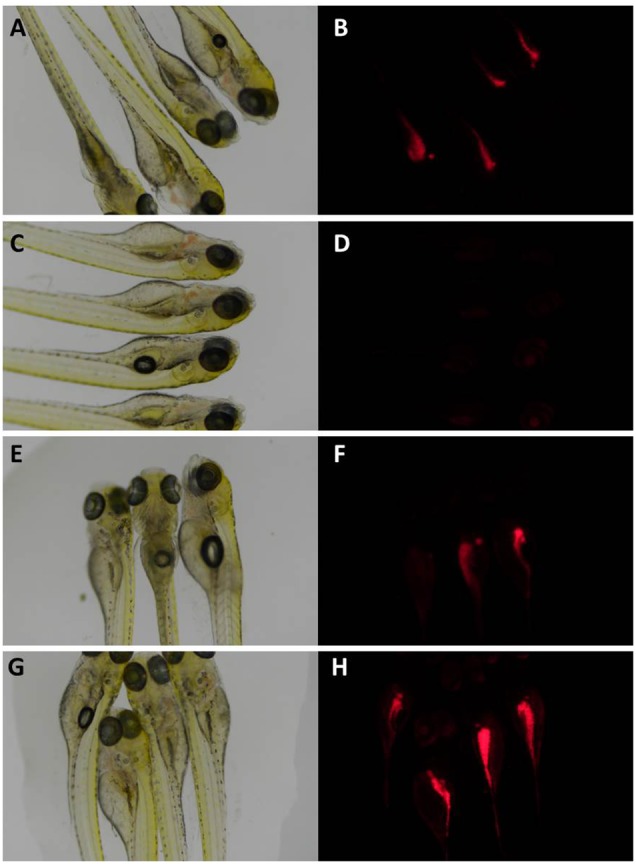
Zebrafish larvae under brightfield **(A,C,E,G)** and fluorescence microscopy **(B,D,F,H)**. In **(A,B)** larvae were exposed only to DMSO, showing a normal lipid buildup, situated mostly among the lower abdomen; in **(C,D)** larvae were exposed to resveratrol, showing a decrease of neutral lipid staining; in **(E,F)** larvae were exposed to extract from strain #91–40; in **(G,H)** larvae were exposed to extract from strain #91-17. These larvae showed a visible decrease and increase in fluorescence when compared to the DMSO treated larvae, respectively. This implies that the extract altered the accumulation of lipids in treated larvae.

**FIGURE 2:**
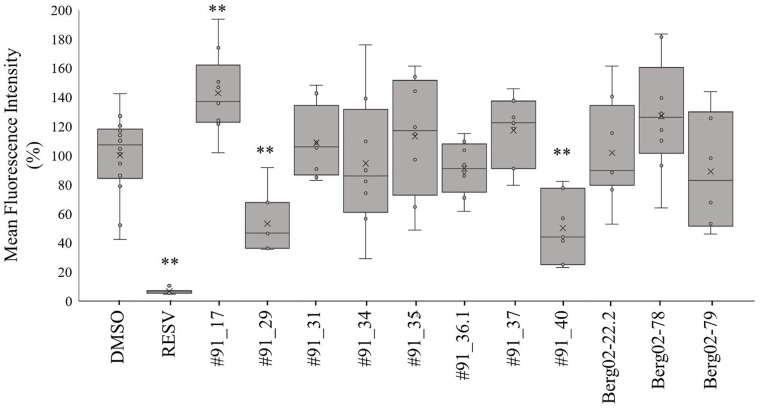
Percentage of fluorescent staining of neutral lipids in zebrafish larvae. Values are presented as mean fluorescence intensity relative to the DMSO group in a box-whisker plot. Statistical differences are represented as asterisks, ^∗∗^*p* < 0.01.

The results of the anti-obesity activity obtained are shown in Figure 1, 2. Larvae incubated with only DMSO, the solvent control of the experiment, showed an intense red fluorescent staining which was considered as the 100% level of lipid content (Figure 1B). As DMSO has low toxicity and does not affect lipid accumulation on zebrafish larvae, as shown by Jones et al. (2008), it is the commonly used solvent in the fish embryo tests (Kais et al., 2013). When larvae were treated with final concentration of 50 μM resveratrol, the positive control of the experiment, a complete absence of red fluorescent staining was visible (Figure 1D, 2) which is indicative of reduction of lipid accumulation.

The zebrafish larvae treated with the majority of extracts did not show different fluorescence levels compared to the DMSO control. However, extracts from two strains, #91_29 and #91_40, reduced significantly the level of fluorescence by 47% and 50% (Figure 1F, 2), respectively. The extract from strain #91_17 induced a significant increase (43%) in fluorescence levels (Figure 1H, 2). Statistical analysis showed that these results are statistically different from the control [*F*_[19,__133]_ = 8.97; *p* < 0.01].

HPLC-HRMS results showed that the extract from strain #91_17 (antifungal activity and increase in neutral lipids) has a high complexity of already identified molecules (Table 5) which are several diketopiperazines and the plant hormone indole acetic acid (IAA). The four diketopiperazines identified in both the extracts from media AB and M016 were cyclo(prolyltyrosyl), cyclo([iso]leucylprolyl), cyclo(phenylalanylprolyl), and cyclo(prolylvalyl). Moreover, four components with the following formulae, C_41_H_60_N_2_O_9_, C_43_H_64_N_2_O_9_, C_22_H_38_N_4_O_5_, and C_22_H_21_N_3_O_3_, were also identified in the extracts. These are not described in the Chapman and Hall Dictionary of Natural Products database.

**Table 5:** Dereplication of the selected active extracts.

Strain ID	Culture medium	Putatively detected components
#91_17	CGY	Cyclo(prolyltyrosyl), cyclo([iso]leucylprolyl), cyclo(phenylalanylprolyl), cyclo(prolylvalyl), 1*H*-indole-3-acetic acid, C_41_H_60_N_2_O_9,_ and C_43_H_64_N_2_O_9_.
#91_17	M016	Cyclo(prolyltyrosyl), cyclo([iso]leucylprolyl), cyclo(phenylalanylprolyl), cyclo(prolylvalyl), 1*H*-indole-3-acetic acid, C_22_H_38_N_4_O_5_ and C_22_H_21_N_3_O_3_
Berg02-22.2	AB	Cyclo([iso]leucylprolyl), Cyclo(phenylalanylprolyl), C_52_H_74_N_10_O_11_, C_35_H_28_N_6_O_8_, C_28_H_41_N_5_O_10_, Gly-Pro-Phe-Pro-Ile peptide, nocardichelin A, nocardichelin B and an undescribed nocardichelin (C_42_H_69_N_5_O_8_).
Berg02-26	IN-CRY	Cyclo([iso]leucylprolyl), cyclo(prolyltyrosyl) C_22_H_21_N_3_O_3_ and C_13_H_14_N_2_O_3_ (coincidence with caerulomycin G.)


**FIGURE 3:**
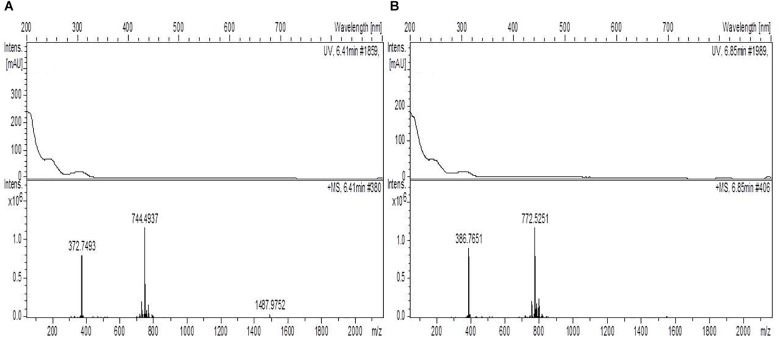
UV and mass spectra of **(A)** nocardichelin A and **(B)** component C_42_H_69_N_5_O_8_ detected in the extract from the AB medium of strain Berg02-22.2. The comparison of the spectra shows the similarities between the two components which suggest that the component C_42_H_69_N_5_O_8_ is a new nocardichelin.

In the extract from the AB medium of strain Berg02-22.2 (antifungal in Duetz and antiparasitic and anticancer activity in EPA vials), both Nocardichelin A (C_40_H_65_N_5_O_8_) and B (C_38_H_61_N_5_O_8_) were found, as well as a component with C_42_H_69_N_5_O_8_ as molecular formula and UV spectrum and ionization pattern similar to both nocardachelins, suggesting that this component is a new nocardachelin not described previously (Figure 3). The peptide Gly-Pro-Phe-Pro-Ile and the diketopiperazines cyclo([iso]leucylprolyl), cyclo(phenylalanylprolyl) were also detected. Furthermore, components with the formulae C_52_H_74_N_10_O_11_, C_35_H_28_N_6_O_8_, C_28_H_41_N_5_O_10_ were found to be present.

The extract from the IN-CRY medium of strain Berg02-26 (antifungal activity) showed the presence of two diketopiperazines and two other compounds. Compound C_22_H_21_N_3_O_3_ seems to be related to the diketopiperazine cyclo(prolyltryrosyl), as the UV spectrum is similar. Component C_13_H_14_N_2_O_3_ matches caerulomycin G in the DNP. However, the UV spectrum is also similar to cyclo(prolyltryrosyl).

## Discussion

The selected strains under study all belong to the *Actinomycetales*, an order that is known for the production of a great number of useful secondary metabolites (Manivasagan et al., 2014; van Keulen and Dyson, 2014; Barka et al., 2016). Although these strains do not belong to the most prolific genera, *Streptomyces* (Ser et al., 2015; Tan et al., 2016; Lee et al., 2018) or *Salinispora* (Jensen et al., 2015), they hold great potential for novel pharmaceutically relevant molecules. The strains here studied were chosen based on previous molecular and screening evidences of bioactive potential (Graca et al., 2013, 2015). As evidenced in Table 1, many of the strains hold key genes to produce secondary metabolites, like polyketide synthases (PKS) and non-ribosomal peptide synthetases (NRPS) and demonstrated antimicrobial bioactivity.

For an overall view of the results obtained, bioactivities and extracts dereplication results are summarized in Table 6.

**Table 6:** Summarized results from the work.

Strain	Afiliation	Lipid assay activity	Bioctive Duetz extracts		Bioctive EPA extracts		Detected components in bioactive EPA extracts
					
			Extract	Bioactivity	Extract	Bioactivity	
#91_17	*Dermacoccus* sp.	-43%	CGY IN-CRY	AF	CGY	AF; CA	Cyclo(prolyltyrosyl), cyclo([iso]leucylprolyl), cyclo(phenylalanylprolyl), cyclo(prolylvalyl), 1H-Indole-3-acetic acid, C41H60N2O9 and C43H64N2O9
			M016		M016	AF; CA	Cyclo(prolyltyrosyl), cyclo([iso]leucylprolyl), cyclo(phenylalanylprolyl), cyclo(prolylvalyl), 1H-Indole-3-acetic acid, C22H38N4O5 and C22H21N3O3
Berg02-22.2	*Gordonia* sp.	NA	AB	AF	AB	TC	Cyclo([iso]leucylprolyl), cyclo(phenylalanylprolyl), C52H74N10O11, C35H28N6O8, C28H41N5O10, the peptide Gly-Pro-Phe-Pro-Ile, nocardichelin A, nocardichelin B and C42H69N5O8
						A2058	
						HepG2	
			IN-CRY	HepG2		HT29	
						MCF7	
						MIAPaCa-2	
Berg02-78	*Gordonia terrae*	NA	AB	AF	N/T		N/T
			IN-CRY	TC			
#91_29	*Microbacterium foliorum*	47%	NA		NA		N/T
#91_40	*Microbacterium foliorum*	50%	IN-CRY	HepG2	N/T		N/T
Berg02-79	*Microbacterium* sp.	NA	R358	MRSA	N/T		N/T
			IN-CRY	HepG2			
Berg02-26	*Micrococcus luteus*	NA	IN-CRY	AF	IN-CRY	AF	Cyclo([iso]leucylprolyl), cyclo(prolyltyrosyl), C22H21N3O3 and C13H14N2O3
			R358			CA	


*AF, A. fumigatus ATCC46645; CA, C. albicans ATCC64124; MRSA, Methicillin-resistant S. aureus MB 5393; TC, T. cruzi Tulahuen C4, N/T, Not tested*.

Our small-scale results not only confirmed the bioactive potential seen previously but proved that several of the selected strains possessed a variable repertoire of bioactivities (Table 3). In the small-scale extraction results, *Dermacoccus* sp. strain #91_17 showed activity against *A. fumigatus* but not against *C. albicans*, as had previously been shown (Graca et al., 2015). However, with medium-scale extraction, *C. albicans* bioactivity was restored (Table 4). Bioactive compounds have been discovered in *Dermacoccus* genus. *Dermacoccus nishinomiyaensis* has displayed the capability to produce monensins A and B, which are produced in a classic polyketide pathway (AlMatar et al., 2017). Monensin has been tested as a chelating agent for the treatment of lead poisoning, with promising results in mice (Ivanova et al., 2016). Phenazine-type pigments, dermacozines, have been isolated from *Dermacoccus abyssi*. Dermacozines F and G have been shown to induce moderate cytotoxic against leukemia cell line K562 (Manivasagan et al., 2014). Hence, *Dermacoccus* has shown to be a genus with a good potential biotechnological value. In this study, *Dermacoccus* sp. strain #91_17 did not display any anti-cancer bioactivity but good anti-fungal activity against *C. albicans* and *A. fumigatus*. Dereplication of the medium-scale extracts pointed to the presence of several bioactive diketopiperazines, the plant hormone IAA and some unidentified molecules (Table 5). The diketopiperazine cyclo(prolyltyrosyl) in the form of (3S, 8aS) can have antibacterial and cytotoxic effects (Bycroft et al., 1988). Cyclo(phenylalanylprolyl) can show different configurations, with one configuration (3R, 8aS) showing some phytotoxic activity and another (3S, 8aS) showing broad antibacterial activity and gastrointestinal cell maturation enhancing activity (Blunt and Munro, 2008). Cyclo(prolylvalyl) is an diketopiperazine antibiotic, with a wide spectrum of activity, with the form (3R, 8aR) active against *Vibrio anguillarum* and the (3S, 8aS) showing a broad antibacterial spectrum (Bycroft et al., 1988). In fact, *Dermacoccus* sp. strain #91_17 demonstrated activity against *V. anguillarum* in a previous study (Graca et al., 2015).

*Gordonia* sp. Berg02-22.2 and *Gordonia terrae* Berg02-78 possess at least one PKS-I gene and showed activity against *Bacillus subtilis* in the previous studies (Graca et al., 2013). Neither *Gordonia* did show any activity against any of the Gram-positive strains tested in our study (although *B. subtilis* was not tested) but, instead, displayed activity against *A. fumigatus* (Table 3). However, antifungal activity of EPA extracts from Berg02-22.2 and Berg02-78 was not observed. Different culture conditions such as the ones in Duetz and EPA can influence the production of bioactive metabolites (Wei et al., 2010). Yet, special relevance can be attributed to strain Berg02-22.2, as these extracts demonstrated anti parasitic and several different anti-cancer activities. Several bioactive capacities of *Gordonia* spp. were referred by Sowani et al. (2018), namely a great antimicrobial capacity of a strain of *G. terrae* isolated from a sponge (Elfalah et al., 2013). Furthermore, Sowani et al. (2018) also reported the various compounds already known to be produced by *Gordonia* genus. These include circumcin A, kurasoin B, soraphinol C; bendigole A, canthxanthin, c-carotene, glycolipids, peptidolipids, and exopolysaccharides. Our work also evidenced the anti-parasitic bioactivity of *Gordonia* sp. Berg02-22.2 against *T. cruzi*. *G. terrae* was isolated from the gut of a *Triatomore* sp. (Gumiel et al., 2015), which is the insect vectors of *T. cruzi* (Lent and Wygodzinsky, 1979). This actinobacterium along with other bacteria present in the gut microbiota of this insect are believed to play a role in the epidemiology of Chagas disease by competing with *T. cruzi* (Gumiel et al., 2015). Moreover, Davila et al. (2011) showed that immunized rats with *G. bronchialis* and challenged with *T. cruzi* had a reduction of parasitemia in offspring. The rat’s immunological system was activated with increased levels of interferons and reduced levels of interleukins. *Gordonia* is, thus, a genus with an extensive biotechnological value. The dereplication of the extract of *Gordonia* sp. strain Berg02-22.2 points to the presence of both nocardichelin A and B, as well as a novel nocardichelin. Both nocardichelin A and B were first isolated from a strain from the genus *Nocardia* and were shown to strongly inhibit human cell lines from gastric adenocarcinoma, breast carcinoma, and hepatocellular carcinoma (Schneider et al., 2007). The peptide Gly-Pro-Phe-Pro-Ile was also detected. This peptide is the result of a pancreatic digestion of β-casein and has been described as a competitive inhibitor for cathepsin B which is upregulated in some cancers (Lee and Lee, 2000). The diketopiperazine cyclo(phenylalanylprolyl) was also detected. Furthermore, components with the formulae C_52_H_74_N_10_O_11_, C_35_H_28_N_6_O_8_, C_28_H_41_N_5_O_10_ were detected in the extract but are of an unknown nature.

Species of the genus *Microccocus* produce pigmented colonies which are sources of carotenoid pigments. Rostami et al. (2016) showed that carotenoid pigments produced by *Microccocus roseus* proved to have some antioxidant, antitumor and potent antibacterial activities, in particular against Gram-positive bacteria. *M. luteus* has very few genes associated with secondary metabolism and possesses one of the smallest genomes from the phylum *Actinobacteria*, comprised of a single circular chromosome with 2.5 Mb in size (Young et al., 2010). Bacteria with genome sizes below 3 Mb usually have fewer or none secondary metabolism genes, while above 5 Mb, there appears to exist a linear correlation between genome size and the presence of these genes (Donadio et al., 2007). This may imply a reduced capacity for the production of bioactive molecules for microorganisms like *M. luteus*. Nevertheless, in both our small-scale and medium-scale extractions, *M. luteus* Berg02-26 proved to produce anti-fungal bioactive molecules and no hepatotoxicity. The dereplication of the extract pointed to the presence of one bioactive diketopiperazine, cyclo(prolyltyrosyl). As discussed above, this diketopiperazine in the form of (3S, 8aS) can have antibacterial and cytoxic effects. Several unidentified molecules were also detected, with compound C_13_H_14_N_2_O_3_ showing a coincidence with caerulomycin G in the DNP. Caerulomycin G is a bypridine alkaloids with a range of activities against *E. coli, Aerobacter aerogenes* and *C. albicans* and cytotoxic activity against the cell lines HL-60, A549 (Kim, 2013).

The small-scale extracts from *Microbacterium* sp. strain Berg02-79 displayed activity against methicillin-resistant *Staphylococcus aureus* and in the HepG2 cell line. Previous studies had shown activity against *Bacillus subtilis* (Graca et al., 2013). Curiously, the methicillin-sensitive strain *Staphylococcus aureus* was not affected by the extract. However, in the medium-scale assay no bioactivity was observed. Once again, the culture conditions may justify this different behavior. *Microbacterium foliorum*, strain #91_40, showed only hepatotoxic activity in the small-scale screening. Additionally, in the zebrafish fat metabolism assay, the extract from this strain displayed possible anti-lipid accumulation activity. Similarly, *Microbacterium foliorum* strain #91_29 also displayed this activity. The zebrafish fat metabolism assay was previously used to identify bioactive compounds from marine fungi and plant polyphenols and has the advantage to mirror the complexity of a whole small animal model and to indicate extracts/compounds with physiologically relevant activities (Noinart et al., 2017; Urbatzka et al., 2018). Natural products isolated from different organisms from marine environments were described to have anti-obesity activities (Castro et al., 2016). *Microbacterium* strains have already shown to possess a varied and interesting repertoire of bioactive secondary metabolites. Microbacterins A and B isolated from a deep-sea strain of *Microbacterium sediminis*, are peptaibols that showed significant inhibitory effects against human tumor cell lines like HCT-8, Bel-7402, BGC-823, A549, and A2780 (Liu et al., 2015). Peptaibols are a class of non-ribosomal linear or cyclic peptides. Additionally, several glycoglycerolipids were isolated from *Microbacterium* sp., and the glycoglycerol GGL.2 showed promising antitumor activities (Wicke et al., 2000).

As the Duetz extraction requires only a very small volume of culture, a great variety of growth conditions could be tested. From the 10 media used for culturing the *Actinomycetales*, only extracts from 5 media were bioactive but differently for each strain. These five media (AB, CGY, IN-CRY, M016, and R358) all have very different composition. With strain #91-17, media CGY, IN-CRY, and M016 were the ones that induced more bioactivities. For strain #91-40 it was medium IN-CRY. Berg02-22.2 and Berg02-78 had better secondary metabolite production in media AB and IN-CRY while Berg02-26 and Berg02-79 the production was favored by media IN-CRY and R358. Remarkably, in the maintenance medium (Marine Broth) no bacterium showed bioactivity. These results give further support to the fact that medium composition is crucial for the activation of the metabolic pathways to produce secondary metabolites. Furthermore, the results obtained illustrated quite well the extended biosynthetic potential of single strains through the “One strain/many compounds” approach (Wei et al., 2010).

The dereplication of our extracts revealed the complexity of molecules possibly produced by the bacterial strains. This molecular diversity can justify the various bioactivities (antifungal, anti-cancer, anti-parasitic, and anti-obesity) obtained. Many of the detected molecules likely possess already known bioactivity. This is the case of molecules like nocardichelins, diketopiperazines, and bipyridine alkaloids. Additionally, eight non-identified compounds were also detected. The variety of components encountered proves that the *Actinomycetales* tested are prolific biosynthesizers, reinforcing the bioactive characteristics of this group.

## Ethics Statement

An approval by an ethics committee was not necessary for the presented work, since chosen procedures are not considered animal experimentation according to the EC Directive 86/609/EEC for animal experiments.

## Author Contributions

The design of the experiments were by JS, RU, FV, and OL. The performing of the scientific work by JS, IV, MC, CD, BC, FA, GP-M, IG, JT, JM, and RU. Manusript writing by JS, IV, MC, CD, BC, FA, GP-M, IG, JT, JM, RU, FV, and OL.

## Conflict of Interest Statement

The authors declare that the research was conducted in the absence of any commercial or financial relationships that could be construed as a potential conflict of interest.

## Footnotes

1 www.enzyscreen.com

## References

[B1] AlMatar M. Eldeeb M. Makky E. A. Koksal F. Var I. Kayar B. (2017). Are there any other compounds isolated from *Dermacoccus* spp at All? *Curr. Microbiol.* 74 132–144. 10.1007/s00284-016-1152-3 27785553

[B2] Annang F. Perez-Moreno G. Garcia-Hernandez R. Cordon-Obras C. Martin J. Tormo J. R. (2015). High-throughput screening platform for natural product-based drug discovery against 3 neglected tropical diseases: human African trypanosomiasis, leishmaniasis, and Chagas disease. *J. Biomol. Screen* 20 82–91. 10.1177/1087057114555846 25332350

[B3] Audoin C. Bonhomme D. Ivanisevic J. de la Cruz M. Cautain B. Monteiro M. C. (2013). Balibalosides, an original family of glucosylated sesterterpenes produced by the mediterranean sponge Oscarella balibaloi. *Mar. Drugs* 11 1477–1489. 10.3390/md11051477 23648552 PMC3707155

[B4] Barka E. A. Vatsa P. Sanchez L. Gaveau-Vaillant N. Jacquard C. Meier-Kolthoff J. P. (2016). Taxonomy, physiology, and natural products of actinobacteria. *Microbiol. Mol. Biol. Rev.* 80 1–43. 10.1128/MMBR.00019-15 26609051 PMC4711186

[B5] Bister B. Bischoff D. Strobele M. Riedlinger J. Reicke A. Wolter F. (2004). Abyssomicin C-A polycyclic antibiotic from a marine *Verrucosispora* strain as an inhibitor of the p-aminobenzoic acid/tetrahydrofolate biosynthesis pathway. *Angew. Chem. Int. Ed. Engl.* 43 2574–2576. 10.1002/anie.200353160 15127456

[B6] Blunt J. W. Munro M. H. G. (2008). *Dictionary of Marine Natural Products, with CD-ROM.* Boca Raton: Chapman & Hall/CRC.

[B7] Bycroft B. W. Higton A. A. Roberts A. D. (1988). *Dictionary of Antibiotics and Related Substances.* New York, NY: Chapman and Hall.

[B8] Castro M. Preto M. Vasconcelos V. Urbatzka R. (2016). Obesity: the metabolic disease, advances on drug discovery and natural product research. *Curr. Top. Med. Chem.* 16 2577–2604. 10.2174/1568026616666160415155644 27086785

[B9] Cautain B. de Pedro N. Schulz C. Pascual J. Sousa Tda S. Martin J. (2015). Identification of the lipodepsipeptide MDN-0066, a novel inhibitor of VHL/HIF pathway produced by a new *Pseudomonas* species. *PLoS One* 10:e0125221. 10.1371/journal.pone.0125221 26018559 PMC4445906

[B10] Davila H. Didoli G. Bottasso O. Stanford J. (2011). Maternal immunization with actinomycetales immunomodulators reduces parasitemias in offspring challenged with Trypanosoma cruzi. *Immunotherapy* 3 577–583. 10.2217/imt.11.14 21463197

[B11] Dhakal D. Pokhrel A. R. Shrestha B. Sohng J. K. (2017). Marine rare actinobacteria: isolation, characterization, and strategies for harnessing bioactive compounds. *Front. Microbiol.* 8:1106. 10.3389/fmicb.2017.01106 28663748 PMC5471306

[B12] Donadio S. Monciardini P. Sosio M. (2007). Polyketide synthases and nonribosomal peptide synthetases: the emerging view from bacterial genomics. *Nat. Prod. Rep.* 24 1073–1109. 10.1039/b514050c 17898898

[B13] Duetz W. A. (2007). Microtiter plates as mini-bioreactors: miniaturization of fermentation methods. *Trends Microbiol.* 15 469–475. 10.1016/j.tim.2007.09.004 17920276

[B14] Duetz W. A. Ruedi L. Hermann R. O’Connor K. Buchs J. Witholt B. (2000). Methods for intense aeration, growth, storage, and replication of bacterial strains in microtiter plates. *Appl. Environ. Microbiol.* 66 2641–2646. 10.1128/AEM.66.6.2641-2646.2000 10831450 PMC110593

[B15] Elfalah H. Usup G. Ahmad A. (2013). Anti-microbial properties of secondary metabolites of marine gordonia tearrae extract. *J. Agric. Sci.* 5 94–101. 10.5539/jas.v5n6p94

[B16] Graca A. P. Bondoso J. Gaspar H. Xavier J. R. Monteiro M. C. de la Cruz M. (2013). Antimicrobial activity of heterotrophic bacterial communities from the marine sponge *Erylus* discophorus (Astrophorida, Geodiidae). *PLoS One* 8:e78992. 10.1371/journal.pone.0078992 24236081 PMC3827338

[B17] Graca A. P. Viana F. Bondoso J. Correia M. I. Gomes L. Humanes M. (2015). The antimicrobial activity of heterotrophic bacteria isolated from the marine sponge *Erylus* deficiens (Astrophorida, Geodiidae). *Front. Microbiol.* 6:389. 10.3389/fmicb.2015.00389 25999928 PMC4423441

[B18] Gumiel M. da Mota F. F. Rizzo Vde S. Sarquis O. de Castro D. P. Lima M. M. (2015). Characterization of the microbiota in the guts of *Triatoma brasiliensis* and *Triatoma pseudomaculata* infected by *Trypanosoma cruzi* in natural conditions using culture independent methods. *Parasit. Vectors* 8 245. 10.1186/s13071-015-0836-z 25903360 PMC4429471

[B19] Hruby A. Hu F. B. (2015). The epidemiology of obesity: a big picture. *Pharmacoeconomics* 33 673–689. 10.1007/s40273-014-0243-x 25471927 PMC4859313

[B20] Imhoff J. F. Labes A. Wiese J. (2011). Bio-mining the microbial treasures of the ocean: new natural products. *Biotechnol. Adv.* 29 468–482. 10.1016/j.biotechadv.2011.03.001 21419836

[B21] International Monetary Fund Research Department [IMF]. (2000). *World Economic Outlook, May 2000 : Asset Prices and the Business Cycle.* Washington, D.C: International Monetary Fund.

[B22] Ivanova J. Gluhcheva Y. Dimova D. Pavlova E. Arpadjan S. (2016). Comparative assessment of the effects of salinomycin and monensin on the biodistribution of lead and some essential metal ions in mice, subjected to subacute lead intoxication. *J. Trace Elem. Med. Biol.* 33 31–36. 10.1016/j.jtemb.2015.08.003 26653741

[B23] Jensen P. R. Moore B. S. Fenical W. (2015). The marine actinomycete genus *Salinispora*: a model organism for secondary metabolite discovery. *Nat. Prod. Rep.* 32 738–751. 10.1039/c4np00167b 25730728 PMC4414829

[B24] Jensen P. R. Williams P. G. Oh D. C. Zeigler L. Fenical W. (2007). Species-specific secondary metabolite production in marine actinomycetes of the genus Salinispora. *Appl. Environ. Microbiol.* 73 1146–1152. 10.1128/AEM.01891-06 17158611 PMC1828645

[B25] Jones K. S. Alimov A. P. Rilo H. L. Jandacek R. J. Woollett L. A. Penberthy W. T. (2008). A high throughput live transparent animal bioassay to identify non-toxic small molecules or genes that regulate vertebrate fat metabolism for obesity drug development. *Nutr. Metab.* 5 23. 10.1186/1743-7075-5-23 18752667 PMC2531115

[B26] Kais B. Schneider K. E. Keiter S. Henn K. Ackermann C. Braunbeck T. (2013). DMSO modifies the permeability of the zebrafish (Danio rerio) chorion-implications for the fish embryo test (FET). *Aquat. Toxicol.* 14 229–238. 10.1016/j.aquatox.2013.05.022 23831690

[B27] Kim S.-K. (2013). *Marine Microbiology : Bioactive Compounds and Biotechnological Applications.* Hoboken, NJ: Wiley 10.1002/9783527665259

[B28] Kwon H. C. Kauffman C. A. Jensen P. R. Fenical W. (2006). Marinomycins A-D, antitumor-antibiotics of a new structure class from a marine actinomycete of the recently discovered genus “marinispora”. *J. Am. Chem. Soc.* 128 1622–1632. 10.1021/ja0558948 16448135

[B29] Lam K. S. Veitch J. A. Golik J. Rose W. C. Doyle T. W. Forenza S. (1995). Production and isolation of two novel esperamicins in a chemically defined medium. *J. Antibiot.* 48 1497–1501. 10.7164/antibiotics.48.1497 8557609

[B30] Lee H. S. Lee K. J. (2000). Cathepsin B inhibitory peptides derived from beta-casein. *Peptides* 21 807–809. 10.1016/S0196-9781(00)00212-6 10959001

[B31] Lee L. H. Chan K. G. Stach J. Wellington E. M. H. Goh B. H. (2018). Editorial: the search for biological active agent(s) from actinobacteria. *Front. Microbiol.* 9:824. 10.3389/fmicb.2018.00824 29780365 PMC5946001

[B32] Lent H. Wygodzinsky P. W. (1979). *Revision of the Triatominae (Hemiptera, Reduviidae), and their Significance as Vectors of Chagas’ Disease.* New York, NY: American Museum of Natural History.

[B33] Liu D. Lin H. Proksch P. Tang X. Shao Z. Lin W. (2015). Microbacterins A and B, new peptaibols from the deep sea actinomycete *Microbacterium sediminis* sp. *nov. YLB-*01(T). *Org. Lett.* 17 1220–1223. 10.1021/acs.orglett.5b00172 25675340

[B34] Manivasagan P. Venkatesan J. Sivakumar K. Kim S. K. (2014). Pharmaceutically active secondary metabolites of marine actinobacteria. *Microbiol. Res.* 169 262–278. 10.1016/j.micres.2013.07.014 23958059

[B35] Mehbub M. F. Lei J. Franco C. Zhang W. (2014). Marine sponge derived natural products between 2001 and 2010: trends and opportunities for discovery of bioactives. *Mar. Drugs* 12 4539–4577. 10.3390/md12084539 25196730 PMC4145330

[B36] Mersch-Sundermann V. Knasmuller S. Wu X. J. Darroudi F. Kassie F. (2004). Use of a human-derived liver cell line for the detection of cytoprotective, antigenotoxic and cogenotoxic agents. *Toxicology* 198 329–340. 10.1016/j.tox.2004.02.009 15138059

[B37] Miao V. Davies J. (2010). Actinobacteria: the good, the bad, and the ugly. *Antonie Van Leeuwenhoek* 98 143–150. 10.1007/s10482-010-9440-6 20390355

[B38] Minas W. Bailey J. E. Duetz W. (2000). Streptomycetes in micro-cultures: growth, production of secondary metabolites, and storage and retrieval in the 96-well format. *Antonie Van Leeuwenhoek* 78 297–305. 10.1023/A:1010254013352 11386352

[B39] Miyamoto S. Izumi M. Hori M. Kobayashi M. Ozaki H. Karaki H. (2000). Xestospongin C, a selective and membrane-permeable inhibitor of IP(3) receptor, attenuates the positive inotropic effect of alpha-adrenergic stimulation in guinea-pig papillary muscle. *Br. J. Pharmacol.* 130 650–654. 10.1038/sj.bjp.0703358 10821794 PMC1572115

[B40] Monteiro M. C. de la Cruz M. Cantizani J. Moreno C. Tormo J. R. Mellado E. (2012). A new approach to drug discovery: high-throughput screening of microbial natural extracts against *Aspergillus fumigatus* using resazurin. *J. Biomol. Screen* 17 542–549. 10.1177/1087057111433459 22233645

[B41] Noinart J. Buttachon S. Dethoup T. Gales L. Pereira J. A. Urbatzka R. (2017). A new ergosterol analog, a new bis-anthraquinone and anti-obesity activity of anthraquinones from the marine sponge-associated fungus *Talaromyces* stipitatus KUFA 0207. *Mar. Drugs* 15 E139. 10.3390/md15050139 28509846 PMC5450545

[B42] Obata H. Muryoi N. Kawahara H. Yamade K. Nishikawa J. (1999). Identification of a novel ice-nucleating bacterium of Antarctic origin and its ice nucleation properties. *Cryobiology* 38 131–139. 10.1006/cryo.1999.2156 10191036

[B43] Palomo S. Gonzalez I. de la Cruz M. Martin J. Tormo J. R. Anderson M. (2013). Sponge-derived Kocuria and *Micrococcus* spp. as sources of the new thiazolyl peptide antibiotic kocurin. *Mar. Drugs* 11 1071–1086. 10.3390/md11041071 23538871 PMC3705389

[B44] Pan R. Bai X. Chen J. Zhang H. Wang H. (2019). Exploring structural diversity of microbe secondary metabolites using OSMAC strategy: a literature review. *Front. Microbiol.* 10:294. 10.3389/fmicb.2019.00294 30863377 PMC6399155

[B45] Perez-Victoria I. Martin J. Reyes F. (2016). Combined LC/UV/MS and NMR strategies for the dereplication of marine natural products. *Planta Med.* 82 857–871. 10.1055/s-0042-101763 27002401

[B46] Rojas J. L. Martin J. Tormo J. R. Vicente F. Brunati M. Ciciliato I. (2009). Bacterial diversity from benthic mats of Antarctic lakes as a source of new bioactive metabolites. *Mar. Genom.* 2 33–41. 10.1016/j.margen.2009.03.005 21798170

[B47] Rostami H. Hamedi H. Yolmeh M. (2016). Some biological activities of pigments extracted from Micrococcus roseus (PTCC 1411) and *Rhodotorula glutinis* (PTCC 5257). *Int. J. Immunopathol. Pharmacol.* 29 684–695. 10.1177/0394632016673846 27895288 PMC5806839

[B48] Schneider K. Rose I. Vikineswary S. Jones A. L. Goodfellow M. Nicholson G. (2007). Nocardichelins A and B, siderophores from nocardia strain acta 3026. *J. Nat. Prod.* 70 932–935. 10.1021/np060612i 17536856

[B49] Schulze C. J. Navarro G. Ebert D. DeRisi J. Linington R. G. (2015). Salinipostins A-K, long-chain bicyclic phosphotriesters as a potent and selective antimalarial chemotype. *J. Org. Chem.* 80 1312–1320. 10.1021/jo5024409 25584395

[B50] Ser H. L. Palanisamy U. D. Yin W. F. Abd Malek S. N. Chan K. G. Goh B. H. (2015). Presence of antioxidative agent, Pyrrolo[1,2-a]pyrazine-1,4-dione, hexahydro- in newly isolated *Streptomyces* mangrovisoli sp. nov. *Front. Microbiol.* 6:854. 10.3389/fmicb.2015.00854 26347733 PMC4542459

[B51] Sowani H. Kulkarni M. Zinjarde S. (2018). An insight into the ecology, diversity and adaptations of *Gordonia* species. *Crit. Rev. Microbiol.* 44 393–413. 10.1080/1040841X.2017.1418286 29276839

[B52] Stowe S. D. Richards J. J. Tucker A. T. Thompson R. Melander C. Cavanagh J. (2011). Anti-biofilm compounds derived from marine sponges. *Mar. Drugs* 9 2010–2035. 10.3390/md9102010 22073007 PMC3210616

[B53] Tan L. T. Chan K. G. Lee L. H. Goh B. H. (2016). *Streptomyces* bacteria as potential probiotics in aquaculture. *Front. Microbiol.* 7:79 10.3389/fmicb.2016.00079 PMC4742533 26903962

[B54] Taylor M. W. Radax R. Steger D. Wagner M. (2007). Sponge-associated microorganisms: evolution, ecology, and biotechnological potential. *Microbiol. Mol. Biol. Rev.* 71 295–347. 10.1128/MMBR.00040-06 17554047 PMC1899876

[B55] Urbatzka R. Freitas S. Palmeira A. Almeida T. Moreira J. Azevedo C. (2018). Lipid reducing activity and toxicity profiles of a library of polyphenol derivatives. *Eur. J. Med. Chem.* 151 272–284. 10.1016/j.ejmech.2018.03.036 29626799

[B56] van Keulen G. Dyson P. J. (2014). Production of specialized metabolites by *Streptomyces* coelicolor A3(2). *Adv. Appl. Microbiol.* 89 217–266. 10.1016/B978-0-12-800259-9.00006-8 25131404

[B57] Vaupel J. W. (2010). Biodemography of human ageing. *Nature* 464 536–542. 10.1038/nature08984 20336136 PMC4010874

[B58] Ventola C. L. (2015). The antibiotic resistance crisis: part 1: causes and threats. *P T* 40 277–283. 25859123 PMC4378521

[B59] Wei H. Lin Z. Li D. Gu Q. Zhu T. (2010). [OSMAC (one strain many compounds) approach in the research of microbial metabolites–a review]. *Wei Sheng Wu Xue Bao* 50 701–709. 20687332

[B60] Wicke C. Huners M. Wray V. Nimtz M. Bilitewski U. Lang S. (2000). Production and structure elucidation of glycoglycerolipids from a marine sponge-associated microbacterium species. *J. Nat. Prod.* 63 621–626. 10.1021/np990313b 10843572

[B61] Yoo Kyung L. Jung-Hyun L. Hong Kum L. (2001). Microbial symbiosis in marine sponges. *J. Microbiol.* 39 254–264.

[B62] You W. Henneberg M. (2018). Cancer incidence increasing globally: the role of relaxed natural selection. *Evol. Appl.* 11 140–152. 10.1111/eva.12523 29387151 PMC5775494

[B63] Young M. Artsatbanov V. Beller H. R. Chandra G. Chater K. F. Dover L. G. (2010). Genome sequence of the fleming strain of *Micrococcus luteus*, a simple free-living actinobacterium. *J. Bacteriol.* 192 841–860. 10.1128/JB.01254-09 19948807 PMC2812450

[B64] Zhang J. H. Chung T. D. Oldenburg K. R. (1999). A simple statistical parameter for use in evaluation and validation of high throughput screening assays. *J. Biomol. Screen* 4 67–73. 10.1177/108705719900400206 10838414

